# Glasgow Royal Infirmary

**Published:** 1831-09

**Authors:** 


### HOSPITAL REPORTS.

### GLASGOW ROYAL INFIRMARY.

***Injuries of the Head.*** **From Dr. Perry's Surgical Report.***

Case I. B. C., labourer. At five p.m., when in a state of inebriety, fell from the top of a stair ten feet high: when raised, was quite insensible, and blood issued from right ear. When admitted next day, lay in a comatose state, and, when roused or spoken to in a loud voice, only made some indistinct murmurs. Eyes turgid and pupils slightly dilated, and sluggish; breathing natural; pulse ninety-two, of moderate strength. Seemed sensible to external impressions. No external injury of skull can be detected. Was bled previous to admission.

The head was shaved, cold lotions applied, and afterwards leeches; the bowels were freely emptied. He lay in the state described, almost without change, till the 22d, when he expired, the fourth day after the accident.

*Inspection.* Over both hemispheres of the brain, beneath the dura mater, and at the base of the skull, there existed a large quantity of coagulated blood. The brain was more vascular than natural. The anterior and left middle lobes were extensively and deeply lacerated, the torn parts filled with coagulated blood, and the medullary substance around this of the consistence of pulp. Several ounces of serum beneath the cerebellum, and a little in the lateral ventricles. The squamous portion of the temporal bone and greater wing of the sphenoid were fractured horizontally, while another fracture took a direction along the petrous portion and sella turcica.

Case II. June 6th. John S., aged thirty-five, lies in a state of general insensibility, from which he cannot be roused. Breathing stertorous; pupils, particularly the right, dilated and immoveable; blood issues from right ear and nostrils; extremities cold; pulse sixty-six, weak and irregular. Above superior posterior angle of right parietal bone is a firm swelling, about the size of half a crown; and at outer canthus of both orbits are small puffy tumors. No fracture detected.

This morning was knocked down by a blow from a fist, and fell on right side of body on pavement: when raised, was insensible, in which state he has since remained. Before admission, was visited by a surgeon, and bled. Died fourteen hours after receipt of accident.

*Inspection.* Skull found fractured below tumor on right side of head. Fracture extended from about the centre of the impression made by temporal muscle on parietal bone, to the middle of the squamous portion of the temporal bone, where it divided, one

* Glasgow Medical Journal.

division going downwards and backwards to the middle part of the mastoid process, when it then ran forwards to the meatus auditorius externus; the other division of the fracture ran forwards and inwards, to foramen lacerum anterius. A portion of the parietal bone was comminuted and slightly depressed. The walls of the cranium, particularly the right parietal bone, were unusually thin, not half the thickness of an ordinary skull, and, when examined by the light, unusually diaphanous. Betwixt the parietal bone and dura mater was a layer of coagulated blood, occupying a space of four inches square; the middle artery of the dura mater was ruptured, and a layer of blood effused between the dura mater and tunica arachnoidea, covering the whole of the right hemisphere and a portion of left; about half an ounce at base of brain. The substance of the brain was healthy.

Case III. James Corbett, æt. twenty-five. June 9th. This morning, about four o'clock, was found lying in a state of stupor below a working steam-engine. At seven A.M., three hours after accident, was brought into this house. Scalp on right side of head, extending from middle of superior part of temporal bone to about same part of occiput, is separated from muscles, so as to form a semicircular flap of about two inches deep. Skull is fractured, apparently about posterior inferior angle of parietal bone of this side, but not depressed. The fracture, which is two lines broad, seems to extend over crown of head, and downwards under right temporal muscle. Immediately over left temple is a puffy swelling, and under it apparent depression of the bone. Left eyelids are much ecchymosed; left clavicle is fractured at middle; on posterior lateral part of right side of chest is a severe lacerated wound, about four inches in length, transversely dividing integuments and muscles down to ribs. Lies in a stupid state, but can be easily roused from it, and when so is incoherent, and can give no distinct answer when questioned. Right pupil is dilated, but sensible to light; the state of left cannot be ascertained, from ecchymosis of eyelids. Breathing natural; has had no vomiting; pulse ninety-six, soft. Has bled a good deal from the wounds.

A consultation met at a quarter before nine, when it was resolved to trace the fracture further, and to puncture swelling over left temple, to ascertain the state of the bone. Accordingly, right temporal muscle was divided to about the extent of an inch, when fracture was found extending to base of skull. Through a puncture made in tumor of left side, bone was thought to be depressed. In this state of things, and as no symptoms of compression were present, it was not thought fit to have recourse to any active measure. At two P.M. the consultation held the same opinion, no change having taken place in the symptoms. Had, on admission, a purging enema.

10th. Has made water freely: heat of skin natural; pupils less dilated, and he appears more sensible; a good deal of thirst.

Rather restless, and strait-jacket applied. To have Calomel gr. x. Adhib. Hir. xij. cap. et ft. venesectio si opus.

11th. Two stools from calomel and senna, and three from drop of croton oil at half-past nine last night. Pulse at present seventy-eight; this morning about sixty, and soft; little pain in head; is more sensible; tongue white; no throbbing of carotid or flushing of face. Cont. lot. frig. cap.; mitt. sang. ad ℥xij. si opus.

12th. A drop of croton oil this morning has produced two stools. Says his head is easy; was restless during the night; pulse forty-eight; skin and pupils natural; did not require bleeding; appears more collected. Cont. lot. cap.

13th. Continues nearly free from pain; slept well; thirst less; countenance more natural; mind calm and sensible; pulse forty-eight; tongue white; three stools; wound on side suppurating kindly. Cont.

14th and 15th. Rather improved, but complains a little of his breast and head. Puffy tumor on left temple gone, and no appearance of depression; wound on scalp suppurating; pulse sixty-four, soft and regular. One stool from drop of croton oil; regularly gets out of bed when he voids urine.

18th. Scalp adhering, and the edges separated for about half an inch, leaving a space of the bone bare about the size of a shilling. Pulse eighty; apparently improving; one stool. Rep. Pilula.

19th. Continues free from pain; sleeps well; pulse sixty-eight. Apparently better; two stools. Cont. Pil.

The patient refused to take the medicines prescribed, and left the hospital, his mind somewhat irritable. About two months after, the portion of bone uncovered by the scalp exfoliated, leaving a space fully the size of a shilling, where the pulsations of the brain were distinctly seen: he came back at this time to the hospital, in good health, and acknowledged that at the time he left it he was not fully sensible of what he was doing.

*Remarks.* It is only necessary to place these cases in contrast, to make them interesting, as shewing the difference betwixt compression of the brain and concussion. The first is a well-marked case of concussion, joined with a certain degree of compression: the second, a case of pure compression: and the third, a case of pure concussion, though from the manner the blow appears to have been received, and the extensive fracture, compression might have been expected to exist. The last case shews, also, how little medical or surgical interference is necessary, when the constitution is otherwise good: here nothing was done except keeping the bowels open, and watching carefully the first appearance of inflammatory symptoms, to act vigorously. This was fortunately not necessary.

It may be further remarked that, in the first case, the absence of the stertorous breathing, the regularity and moderate state of the pulse, the ability, to a certain extent, to use the voluntary muscles, without the power of directing them to any object, and the excitability of the involuntary muscles, might have led to the belief that the case was one of concussion; but the blood issuing from the ear, the loss of speech and of mind, indicated, along with this, either extravasation or serious injury of the brain, which was illustrated by the post-mortem examination. While, in the second case, the complete insensibility to external impressions, the stertorous breathing, dilated pupils, irregular pulse, and coldness of the extremities, with the blood issuing from the ear, all immediately following the injury, indicated complete compression of the brain, and consequently the nearly total suspension of the action, of both voluntary and involuntary muscles. In the third case, though the patient lay for some days in a stupid state, he could easily be roused. Though incoherent, the action of the voluntary and involuntary muscles was perfect, as shewn by his exertions, and his getting out of bed when he felt the inclination to void urine.

It will be remarked that, in the two first cases, the patients had been bled previous to admission, immediately after the receipt of the injury. This practice cannot be too severely reprobated, as there is no doubt that, in many cases, it has the effect of sinking the patient irrecoverably. It is true that, in the cases detailed, nothing could have saved the patients, but it might have been otherwise. In the third case, this practice might have been followed by serious consequences.

---

### *Immediate Amputation after Injuries.**

In cases of compound fracture, or extensive laceration of limbs, where the patient is much sunk with the shock, it often becomes a question, requiring both skill and decision on the part of the surgeon, whether immediate amputation should be had recourse to, or that he should wait to see if reaction will take place. If amputation be necessary, and is not forbid by the existence of some other injury that may be supposed to be fatal, I would be much inclined to recommend immediate amputation; as, in such cases, we may wait for a reaction which will never take place. The extensive laceration and bruising, accompanied perhaps with bleeding, keep up the pain and irritation, as well mental as bodily, which still further exhausts the patient; an irritation which may, after all, be speedily removed by the immediate amputation of the lacerated limb. The two following cases illustrate the practice which I am disposed to adopt under such circumstances.

Case I. James Cullen, aged thirty-five; 30th March, half-past nine p.m. Half an hour before admission, was engaged removing a tree from a cart, which slipped over the wheel and struck him with the edge of its thickest end on right leg.

There is great bruising and laceration of integuments above instep, where a broad wound presents itself, extending obliquely

* Ibid.

from a little above outer ankle, nearly seven inches upwards, and about four inches transversely; there is considerable laceration of neighbouring soft parts. About three inches above instep there is an abrupt projection of shaft of tibia, which is much shattered; fibula is also fractured; lost a good deal of blood by oozing; limb cold, with some emphysema inferiorly; pulse feeble, and features sunk; general health previously good. A consultation being instantly called, it was agreed that the limb should be immediately amputated below knee, which was done. He bore the operation well, though previously much sunk, and revived immediately after the operation, declaring himself greatly better before he left the operation table. Both tibia and fibula were found minutely comminuted, and the soft parts lacerated to within four inches of knee.

The patient ultimately did well.

Case II. April 11. James Laughlin, æt. fifty. An hour before admission, left leg and toes of right foot were crushed on a railway by seven loaded waggons passing over him.

Integuments are completely torn away from front of leg down to foot, exposing the deep-seated parts, which are severely bruised and lacerated along the bones; tibia and fibula are fractured and comminuted at their lower third; and the mucles are nearly torn across, leaving the lower portion of the leg and foot attached by a flap, consisting, for the most part, of the deep-seated muscles on back part of leg; all the bones of the tarsus and metatarsus are comminuted. Integuments of toes on right foot are lacerated, and the phalanges of the four greater toes comminuted. Is said to have lost much blood; is sunk and oppressed; has had frequent vomiting; countenance pale; skin cold; pulse feeble; some apparent incoherence. A consultation being instantly called at half-past three P.M., it was agreed that the limb should be taken off immediately, above the knee, which was accordingly done, by the double flap operation. He stood the operation well, although the pulse was so feeble that it could not be numbered, revived after the operation, and in the evening was more cheerful, when the great toe and the three adjoining toes were removed, at their metatarsal articulations. Says the former operation was nothing compared with this. Complains of pain and nausea at stomach. To have two grains of opium.

12th. Appears much revived; pulse 120, weak; heat of skin natural; tongue clean; passed a very restless night; complains much of pain about stomach and chest. Sumat. stat. Bol. Com.

13th. This morning began to sink without any obvious cause; vomits every thing he takes; pulse so weak as not to be numbered; tongue pale; incoherent; to have brandy, *ad libitum.* Died at five next morning.

*Inspection.* On opening the chest, the lungs and heart appeared natural. On removing the left lung, some soft adhesions were observed at its inferior posterior part, and about ten ounces of a reddish coloured sero-purulent fluid were effused into the cavity of the left pleura. The ninth rib was found to have sustained a comminuted fracture, at the distance of an inch from its vertebral connexion. The pleura costalis, for the space of two inches, was thickened, softened, and of a deep red hue; the fluid in the stomach was dark and grumous. No other morbid appearances could be detected.

More cases of a similar nature might be brought forward. On the other hand, I have seen cases where the patients were even less sunk than the last, and not more so than the first, allowed to remain in the hope that reaction might take place; in place of which, they continued to sink till death put a period to their sufferings, as would have been the case with Laughlin in a very few hours. It may be mentioned, that the patients were liberally supplied with wine and spirits, with a view to excite reaction, but the knife proved the best stimulus.

